# Evaluation of Salinity Tolerance Potentials of Two Contrasting Soybean Genotypes Based on Physiological and Biochemical Responses

**DOI:** 10.3390/plants15010010

**Published:** 2025-12-19

**Authors:** Mawia Sobh, Tahoora Batool Zargar, Oqba Basal, Ayman Shehada AL-Ouda, Szilvia Veres

**Affiliations:** 1 Faculty of Agricultural and Food Sciences and Environmental Management, Institute of Applied Plant Biology, University of Debrecen, Böszörményi út 138, 4032 Debrecen, Hungary; 2 Department of Field Crops, Faculty of Agriculture, Damascus University, Damascus 10001, Syria

**Keywords:** soybean, salinity tolerance, physiological traits, biochemical traits, ion homeostasis, hydroponics

## Abstract

Salinity stress is a major abiotic constraint limiting soybean (*Glycine max* L.) productivity in saline–alkali soils; however, the physiological and biochemical mechanisms underlying genotypic tolerance remain poorly understood. This study aimed to identify key traits that underpin salinity tolerance and can inform breeding and agronomic strategies to enhance soybean performance under saline conditions. Two contrasting soybean genotypes, YAKARTA and POCA, were exposed to 25, 50, 75, and 100 mM NaCl from the first to the fourth trifoliate stage (V1–V4) under controlled conditions for 30 days. YAKARTA maintained higher relative water content (75.51% vs. 66.97%), stomatal conductance (342 vs. 286 mmol H_2_O m^−2^ s^−1^), proline (6.15 vs. 4.36 µmol g^−1^ fresh weight), K^+^/Na^+^ ratio (61.8 vs. 32.2), and H_2_O_2_ (833.8 vs. 720.2 µmol g^−1^ fresh weight) compared with POCA, whereas POCA exhibited elevated solute leakage (87.1% vs. 79.21%), malondialdehyde (122 vs. 112 µg g^−1^), and ascorbic acid (334 vs. 293 µg g^−1^), indicating greater sensitivity. At 100 mM NaCl, relative water content, stomatal conductance, K^+^/Na^+^ ratio, and H_2_O_2_ declined by 44.5%, 81.9%, 99.8%, and 49.5%, respectively, while proline, solute leakage, malondialdehyde, and ascorbic acid increased by 56-, 1.27-, 11.6-, and 1.68-fold, respectively. The contrasting physiological and biochemical responses between these genotypes highlight key traits, such as relative water content, stomatal conductance, proline accumulation, malondialdehyde content, and the K^+^/Na^+^ ratio, as promising potential markers associated with salinity tolerance in soybean. These findings provide a foundational understanding that can guide future research to validate these markers across a wider genetic pool and under field conditions.

## 1. Introduction

Soybean (*Glycine max* (L.) Merrill) is the most widely harvested seed legume globally and a major source of affordable protein (36–40%) and vegetable oil (18–24%) [1]. Due to its excellent nutritional profile and health benefits, it plays a vital role in human nutrition, animal feed, and biofuel production, with approximately 85% of the global yield utilized across these sectors [2]. Additionally, soybean serves as an effective intercrop [3], enhancing soil fertility and sustainability in agricultural systems.

Despite its global importance, soybean is a salt-sensitive crop, making it particularly vulnerable to soil salinization, a growing threat to agricultural productivity. Salinity is a major environmental challenge that significantly hinders crop growth and productivity worldwide [4,5]. It disrupts early seedling growth and interferes with various physiological and biochemical processes, depending on the plant’s developmental stage and genetic background [6]. For instance, Chen et al. [7] reported that electrical conductivity (EC) levels of 5 dS m^−1^ or higher inhibit soybean germination, while levels of 7.3–9.6 dS m^−1^ can cause complete stand loss in salt-sensitive and moderately tolerant genotypes.

Soil salinity is projected to become even more severe, with nearly 50% of arable land expected to be affected by 2050 [8,9]. The global salinization of land is increasing at a rate of approximately 10% per year [10], which poses a significant threat to global food security, as the world will need to produce 70% more food to feed a population of 9.3 billion by 2050 [11]. This rise in salinity is driven by several factors, including climate change, improper irrigation, high evapotranspiration, poor drainage practices, excessive fertilizer usage, and insufficient water management [12].

Salinity reduces water uptake, alters ionic balance, and impairs vital physiological functions. Excess sodium (Na^+^) disrupts potassium (K^+^) uptake due to shared transport pathways, resulting in reduced K^+^ concentrations and impaired enzyme activity, stomatal conductance, transpiration, and photosynthesis, while promoting the accumulation of compatible solutes such as proline [13,14,15]. Salinity also triggers osmotic and oxidative stress, leading to the accumulation of reactive oxygen species (ROS) such as superoxide (O_2_^−^), hydrogen peroxide (H_2_O_2_), and hydroxyl radicals (OH^−^), which damage cellular components [16,17]. Sodium chloride-induced salinity results in both osmotic and ionic stress, with sodium and chloride buildup causing ion toxicity and rapid osmotic shock [18].

Plants have evolved various mechanisms to cope with stress, including the accumulation of osmoprotectants like proline, regulation of Na^+^/K^+^ homeostasis, and activation of antioxidant defenses [14,15]. However, tolerance varies significantly across genotypes and developmental stages, particularly during early vegetative growth and flowering [19]. Genotypic differences in physiological traits under controlled stress conditions have also been observed [20]. Since seedling vigor and tolerance at the early vegetative stage largely determine plant establishment, subsequent biomass accumulation, and ultimately reproductive success, studying these early responses provides key insights into the biology of salinity tolerance. Breeding salt-tolerant soybean varieties is a promising strategy to mitigate yield losses in saline environments, but it requires comprehensive screening tools that integrate physiological and biochemical markers.

Despite numerous studies on salinity stress in soybeans, a lack of data remains on comparative physiological and biochemical responses among specific genotypes under controlled hydroponic conditions. For example, Mannan et al. [21] and Essa [22] showed reductions in biomass and water content under increasing NaCl, while Amirjani [23] and Wu et al. [24] highlighted the importance of ion balance in salt tolerance. Other studies, such as those by Das et al. [25] and Sadak et al. [26], emphasized the role of proline accumulation, whereas Noreen et al. [27] and Razmi et al. [28] investigated the role of antioxidants like ascorbic acid and enzymatic ROS detoxification.

However, few studies have integrated these physiological and biochemical markers comprehensively across underexplored genotypes in a hydroponic system. Many past studies have focused on agronomic performance under field conditions [24,26] or on molecular-level responses without linking them to observable physiological traits. Moreover, limited attention has been paid to integrating key biochemical markers, such as hydrogen peroxide, malondialdehyde, and ascorbic acid, alongside physiological indicators like relative water content and stomatal conductance. By combining these indicators, it is possible to bridge molecular insights with whole-plant responses, providing a more mechanistic understanding of salinity tolerance.

To address this gap, the present study evaluates two soybean genotypes, YAKARTA and POCA, which have not been widely compared in salinity research. Previous studies have shown that soybean responses to NaCl-induced salinity are typically evaluated within a 25–100 mM range to simulate mild to severe stress conditions [22,23,26]. Accordingly, this range was selected to induce measurable physiological and biochemical responses without causing complete plant mortality.

We hypothesize that the soybean genotypes YAKARTA and POCA exhibit differential physiological and biochemical responses to salinity stress under hydroponic conditions, and that these differences can be used to identify reliable screening markers for salt tolerance. Therefore, the objectives of this study are to:

1. Evaluate and compare the effects of salinity on key physiological traits, including relative water content, stomatal conductance, membrane stability index, and K^+^/Na^+^ ratio;
2. Assess changes in biochemical markers such as proline content, hydrogen peroxide (H_2_O_2_), malondialdehyde (MDA), and ascorbic acid (AsA) under salt stress;
3. Examine the interrelationships among physiological and biochemical traits under salinity through correlation analysis to identify potential markers associated with salt tolerance;
4. Apply principal component analysis (PCA) to integrate physiological and biochemical data for distinguishing genotype-specific responses and identifying key contributing traits to salt tolerance;
5. Identify genotype-specific responses that may inform the selection and breeding of salt-tolerant soybean cultivars.

The results are expected to provide a physiological and biochemical framework for screening salinity tolerance, offering reliable biological markers that can accelerate the identification of resilient genotypes and ultimately support breeding strategies aimed at improving soybean performance in salt-affected environments.

## 2. Materials and Methods

### 2.1. Plant Material and Genotypes

Two soybean (*Glycine max* L. Merrill) genotypes (YAKARTA and POCA) were used in this experiment. Seeds were obtained from the National Soybean Germplasm Bank, Hungarian Research Institute of Plant Breeding and Crop Production (HIPP), which maintains both lines as part of its registered germplasm collection. According to HIPP’s classification, these genotypes display contrasting performance during preliminary evaluations under abiotic stress conditions, with YAKARTA generally showing stronger vegetative vigor and POCA exhibiting relatively weaker stress responses. Although published characterizations of these specific lines remain limited, the use of physiologically contrasting soybean genotypes is a well-established and widely accepted approach for dissecting salinity tolerance mechanisms [22,29,30,31]. Therefore, YAKARTA and POCA were selected intentionally to represent divergent response patterns and enable a clearer interpretation of salinity-induced physiological and biochemical alterations.

### 2.2. Seed Preparation and Germination

Before sowing, seeds were surface sterilized in a 6% (*v*/*v*) hydrogen peroxide (H_2_O_2_) solution for 20 min to eliminate microbial contaminants. They were then thoroughly rinsed with deionized water. Germination was initiated by placing the seeds geotropically between moistened filter papers and incubating them at 24 °C. After five days, uniformly germinated seedlings (at the VE stage) were selected for transplantation.

### 2.3. Hydroponic Growth Conditions

Seedlings were transferred into 1.7 L plastic pots (four plants per pot) containing a modified dicot nutrient solution. The composition of the solution was as follows: 0.7 mM K_2_SO_4_, 0.5 mM MgSO_4_, 0.1 mM KH_2_PO_4_, 0.1 mM KCl, 0.5 µM MnSO_4_, 0.5 µM ZnSO_4_, 10 µM H_3_BO_3_, 0.2 µM CuSO_4_, and 2.0 mM Ca (NO_3_) _2_. Iron was supplied as 10^−4^ M Fe-EDTA [32]. The pH of the nutrient solution was maintained between 5.8 and 6.2 and adjusted every three days to ensure optimal nutrient availability. The entire nutrient solution was replaced with fresh solution every three days. Plants were cultivated in a controlled environment growth chamber under standardized conditions: 24/18 °C (±1 °C) day/night temperature, a 10/14 h light/dark photoperiod, relative humidity of 45%, and a photosynthetic photon flux density of 300 μmol m^−2^ s^−1^.

### 2.4. Salinity Stress and Treatment Application

Salinity stress was applied two weeks after sowing, when soybean plants had developed their first trifoliate leaves (early vegetative stage). Seedlings were transplanted into hydroponic nutrient solution, and sodium chloride (NaCl) was added at concentrations of 25, 50, 75, and 100 mM NaCl to simulate increasing levels of salt stress. A control group (0 mM NaCl) was maintained under optimal growth conditions without added salt. These salinity levels were selected based on established protocols for screening soybean under salt stress, which have been shown to elicit a progressive physiological response from moderate osmotic stress to severe ion toxicity [30,33,34,35]. Using this gradient allows assessment of early and severe stress responses, facilitating identification of threshold levels for salinity tolerance. Additional studies have also applied similar or broader NaCl ranges, confirming the suitability of these concentrations for hydroponic experiments [36,37]. To minimize osmotic shock, NaCl was introduced gradually over six hours for each concentration. Salinity treatments were maintained continuously for 30 days, with final NaCl concentrations verified using a calibrated conductivity meter. At the end of the treatment period, plants were harvested for morphological, physiological, and biochemical assessments to evaluate genotype-specific responses to salinity stress.

### 2.5. Experimental Design

The experiment was arranged in a factorial design combining two soybean genotypes and five NaCl concentrations (2 × 5) in a completely randomized design (CRD). Each treatment was replicated three times, with each replicate consisting of one pot containing four plants. In total, the experiment included 30 pots (2 genotypes × 5 treatments × 3 replicates), representing 120 individual plants.

### 2.6. Data Collection

#### 2.6.1. Physiological Measurements

##### Shoot Dry Weight (g)

Shoot dry weight (g) was recorded 30 days after salinity treatment. Shoots were harvested, oven-dried at 70 °C for four days until a constant weight was achieved and then weighed using an analytical balance to determine dry biomass.

##### Measurement of Leaf Relative Water Content (RWC%)

Trifoliate leaf samples were collected at the end of the experiment from randomly selected plants and were weighed to determine fresh weight (FW). After rehydration in water for 4 h, the samples were weighed again to obtain turgid weight (TW). Finally, the samples were dried at 70 °C for 24 h. and weighed to get constant dry weight (DW). Relative water content values were calculated using the formula [38].

RWC (%) = [(FW − DW)/(TW − DW)] × 100

##### Stomatal Conductance Measurements (g_s_, mmol H_2_O m^−2^ s^−1^)

Stomatal conductance (gs) was measured using an AP4 porometer (Delta-t devices, Cambridge, UK). Measurements were taken from the uppermost fully expanded and healthy leaves of three plants per genotype and treatment. To minimize diurnal variation and ensure consistency, data collection was conducted between 9:00 and 11:00 AM under ambient light and temperature conditions. This time window was selected to capture active stomatal function while avoiding fluctuations associated with early morning and midday stomatal behavior.

#### 2.6.2. Biochemical Measurements

##### Measurement of Proline Content (Prol., µmol g^−1^ Fresh Weight)

Using a technique established by Bates et al. [39], proline was extracted from 0.2 g of leaf tissues homogenized in 4 mL of 3% aqueous sulfosalicylic acid. The supernatant (2 mL) was combined with 2 mL of ninhydrin and 2 mL of glacial acetic acid after centrifugation at 10,000 rpm. This mixture was then heated for an hour at 100 °C. Toluene (4 mL) was used to extract the reaction mixture, and 590 nm was used to measure the absorbance. The standard curve was used to compute the final proline concentration.

##### Measurement of Membrane Integrity (Solute Leakage) (%)

Solute leakage (SL) was measured as described by Leopold et al. [40]. Plant material (0.5 g) was washed with deionized water, placed in tubes with 10 mL of deionized water and left on a shaker for 3 hrs. at 25 °C. Subsequently, the initial absorbance of the solution (A1) was determined at an optical density of 273 nm. The samples were then boiled in a boiling bath at 100 °C for 20 min, and the final absorbance of the solution (A2) was determined at the same optical density. The percentage of the solute leakage was measured according to the following formula.

SL (%) = (A1/A2) × 100

##### Lipid Peroxidation Assay (µmol g^−1^ Fresh Weight)

Membrane lipid peroxidation was assessed using the method described by Heath and Packer [41]. Fresh leaf tissue (0.2 g) was homogenized in 5 mL of 0.1% Trichloroacetic acid (TCA) and centrifuged at 10,000 rpm. The supernatant was mixed with a solution of 20% TCA and 0.5% thiobarbituric acid in a 1:4 (*v*/*v*) ratio and heated at 90 °C for 30 min. The amount of oxidized malondialdehyde (MDA) was calculated using the following formula:

MDA (M) = (A_532_ − A_600_)/(ε × b)

where A_532_ and A_600_ are the absorbance values at 532 nm and 600 nm, respectively, ε is the molar extinction coefficient for the MDA-TBA complex (1.55 × 10^5^ M^−1^ cm^−1^), and b is the path length of the cuvette (1 cm).

##### Measurement of Na^+^ and K^+^ Ratio

Dried leaf (1.0 g) tissue was digested in a mixture of HClO_4_ and HNO_3_ until the solution turned clear. Digested material was made to 100 mL by adding distilled water and read on a flame photometer for quantification of Na^+^ and K^+^ [42], and then the ratio of K^+^ to Na^+^ was computed.

##### Measurement of Ascorbic Acid Content (AsA, µg g^−1^)

The concentration of ascorbic acid in each genotype, treatment and replicate was estimated according to the method of DePinto et al. [43]. The absorbance was measured at 525 nm using a spectrophotometer, and a standard curve was used for calculation.

##### Measurement of Hydrogen Peroxide (H_2_O_2_, µmol g^−1^ Fresh Weight)

Hydrogen peroxide content in leaf samples was determined following the method of Velikova et al. [44]. The absorbance was measured at 390 nm, and hydrogen peroxide content was calculated using a calibration curve generated from known H_2_O_2_ concentrations.

### 2.7. Statistical Analysis

All recorded data were analyzed using MSTAT-C (version 8.1.2) software. A factorial analysis of variance (ANOVA) was conducted to evaluate the effects of genotype, salinity level, and their interaction. Mean comparisons were performed using the Least Significant Difference (LSD) test at the 1% significance level. The coefficient of variation (CV%) was calculated to evaluate the consistency of and variability in the data across replicates. To assess the relationships among the measured traits, Pearson’s correlation analysis was performed. In addition, principal component analysis (PCA) was conducted to reduce data dimensionality and to visualize patterns of variation among genotypes and salinity treatments.

## 3. Results

### 3.1. Shoot Dry Weight (g)

**Figure 1:**
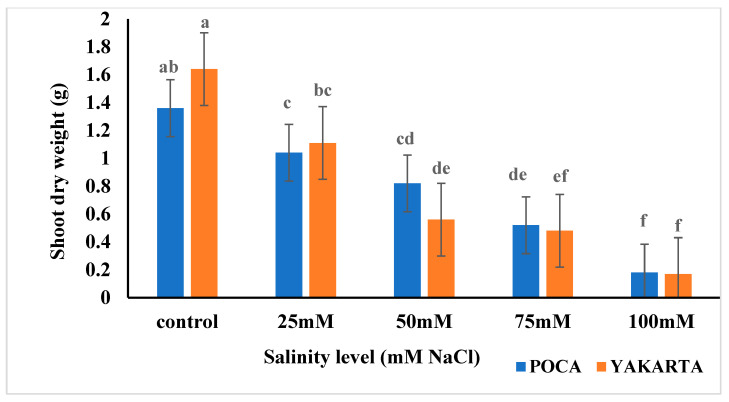
Effect of NaCl-induced salinity stress on shoot dry weight (g) of two soybean genotypes under hydroponic conditions (n = 3, ±s.e.). Different lowercase letters indicate significant differences (*p* ≤ 0.01) for the interactions between salinity levels and genotypes.

Results revealed no significant differences in shoot dry weight between the two soybean genotypes overall, but significant effects (*p* ≤ 0.01) were observed for both salt levels and the genotype by salinity interaction. Shoot dry weight decreased with increasing salinity. Under control and 25 mM NaCl, YAKARTA maintained higher values than POCA (1.64 vs. 1.36 g and 1.11 vs. 1.04 g, respectively). At 50 mM NaCl, however, POCA (0.82 g) exhibited a significantly higher shoot dry weight than YAKARTA (0.56 g), highlighting a differential response at this salt level. Both genotypes experienced substantial reductions in shoot dry weight at 75 and 100 mM NaCl, with POCA decreasing to 0.52 g at 75 mM and reaching its lowest value (0.17 g) at 100 mM, while YAKARTA declined to 0.48 g at 75 mM and 0.18 g at 100 mM (Figure 1 and Figure S1).

### 3.2. Relative Water Content (%)

Statistical analysis revealed significant main effects of genotype (*p* ≤ 0.01), salt concentration (*p* ≤ 0.01), and their interaction (genotype × salt level, *p* ≤ 0.01) on relative water content (RWC). Within the YAKARTA genotype, RWC significantly decreased with increasing salt concentration, from 94.25% in the control to 57.83% at 100 mM NaCl. A similar decreasing trend was observed for POCA, with RWC dropping from 89.83% (control) to 44.35% at 100 mM NaCl. At each salt level, YAKARTA consistently showed higher RWC than POCA, and the difference was statistically significant at 50 mM, 75 mM, and 100 mM NaCl. The highest RWC values were recorded in both genotypes under control conditions, while the lowest was found in POCA at 100 mM (Table 1). These findings indicate that although both genotypes were affected by salinity, YAKARTA maintained higher water content under stress conditions than POCA, suggesting a possible difference in osmotic adjustment or membrane stability.

### 3.3. Stomatal Conductance (mmol H_2_O m^−2^ s^−1^)

**Figure 2:**
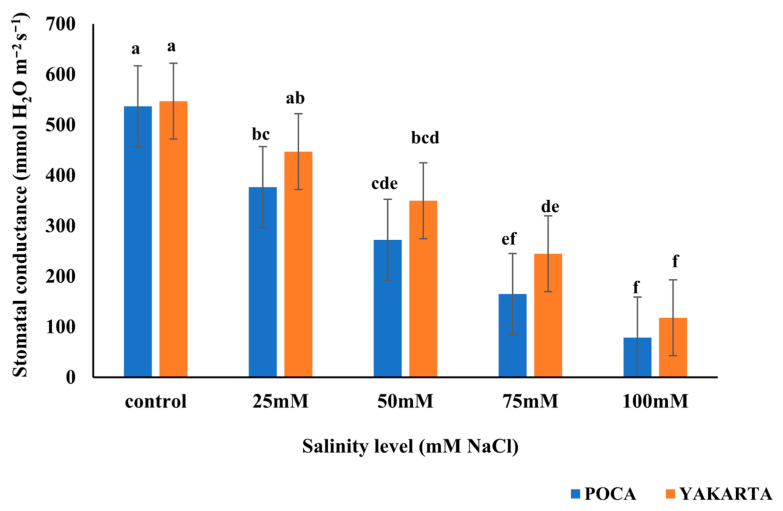
Effect of NaCl-induced salinity stress on stomatal conductance (mmol H_2_O m^−2^ s^−1^) of two soybean genotypes under hydroponic conditions (n = 3, ±s.e.). Different lowercase letters indicate significant differences (*p* ≤ 0.01) for the interactions between salinity levels and genotypes.

Statistical analysis revealed significant effects (*p* ≤ 0.01) of genotype, salinity level, and their interaction on stomatal conductance (g_s_). In general, g_s_ decreased significantly with increasing salt concentrations in both genotypes. Within the YAKARTA genotype, g_s_ declined from 547.00 mmol H_2_O m^−2^ s^−1^ under control conditions to 118.00 mmol H_2_O m^−2^ s^−1^ at 100 mM NaCl. Similarly, POCA showed a reduction from 536.70 mmol H_2_O m^−2^ s^−1^ (control) to 78.67 mmol H_2_O m^−2^ s^−1^ (100 mM NaCl). At lower salt levels (0 and 25 mM NaCl), no significant differences were observed between genotypes. However, under moderate to high salt stress (50–100 mM NaCl), YAKARTA maintained significantly higher g_s_ values than POCA (Figure 2). These results suggest that although stomatal conductance is inhibited by salinity in both genotypes, YAKARTA exhibits better stomatal regulation under stress, which may contribute to improved water-use efficiency and gas exchange maintenance under saline conditions.

### 3.4. Proline Content (µmol g^−1^ Fresh Weight)

Results revealed highly significant effects (*p* ≤ 0.01) of genotype, salinity level, and their interaction on proline accumulation. Proline content increased significantly with rising NaCl concentrations in both genotypes. Within the YAKARTA genotype, proline rose sharply from 0.25 µmol g^−1^ fresh weight under control conditions to 15.3 µmol g^−1^ fresh weight at 100 mM NaCl. In POCA, proline also increased, but to a lesser extent, reaching 12.8 µmol g^−1^ fresh weight at 100 mM. At lower salt levels (0, 25, and 50 mM NaCl), there were no significant differences in proline content between treatments or genotypes. However, under higher salt levels (75 and 100 mM NaCl), YAKARTA exhibited significantly higher proline accumulation than POCA (Table 2). These results suggest that YAKARTA responds to salt stress with a more pronounced increase in proline accumulation, a known osmoprotectant, indicating its potentially stronger adaptive response under saline conditions.

### 3.5. Membrane Integrity (Solute Leakage) (%)

Results indicated significant effects (*p* ≤ 0.01) of genotype, salinity level, and their interaction on solute leakage. Across treatments, POCA consistently exhibited a higher percentage of solute leakage than YAKARTA, indicating greater membrane damage under salt stress. In both genotypes, solute leakage increased progressively with rising salinity levels. YAKARTA showed a significant increase from 63.4% in the control to 91.2% at 100 mM NaCl, while POCA increased from 81.6% to 94.2% across the same range. At all salt levels, except 100 mM, YAKARTA had significantly lower solute leakage than POCA, suggesting better membrane integrity. The lowest leakage was observed in YAKARTA under control conditions, while the highest was recorded in POCA at 100 mM NaCl. (Table 3). The stronger membrane stability in YAKARTA may be attributed to its higher relative water content and greater proline accumulation under salt stress, both of which support cellular homeostasis and reduce ion-induced damage.

### 3.6. Malondialdehyde Content (µmol g^−1^ Fresh Weight)

**Figure 3:**
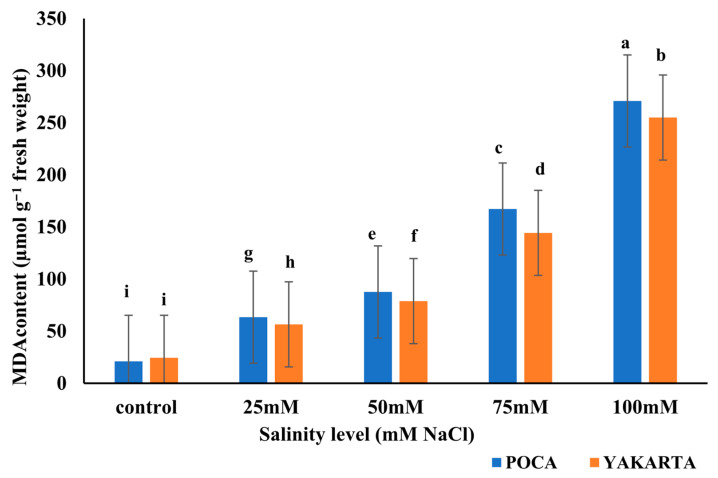
Effect of NaCl-induced salinity on MDA (µmol g^−1^ fresh weight) of two soybean genotypes under hydroponic conditions (n = 3, ±s.e.). Different lowercase letters indicate significant differences (*p* ≤ 0.01) for the interactions between salinity levels and genotypes.

The statistical analysis results revealed highly significant effects (*p* ≤ 0.01) of genotype, salinity level, and their interaction on MDA content. MDA, a biomarker of lipid peroxidation and membrane damage, increased progressively with rising salinity in both genotypes. The analysis of variance revealed a significant main effect of genotype on MDA content (*p* < 0.01). Averaged across all salinity levels, POCA exhibited significantly higher MDA levels (122.0 µmol g^−1^ fresh weight) compared to YAKARTA (112 µmol g^−1^ fresh weight), indicating greater inherent oxidative stress or membrane damage. This overall trend is reflected in Figure 3, where POCA shows higher MDA values than YAKARTA at most individual salinity levels. At the control level, both genotypes recorded the lowest MDA values, with no significant difference between them (24.40 µmol g^−1^ fresh weight in YAKARTA; 21.05 µmol g^−1^ fresh weight in POCA). MDA content increased significantly at higher salt levels, reaching the highest values at 100 mM NaCl, with POCA recording 270.90 µmol g^−1^ fresh weight and YAKARTA 255.00 µmol g^−1^ fresh weight (Figure 3). These findings indicate that membrane lipid peroxidation was strongly induced by NaCl stress in both genotypes, but more severely in POCA. The results are consistent with the observed differences in relative water content and proline accumulation, both of which are associated with oxidative stress protection.

### 3.7. The Potassium-to-Sodium Ratio

Statistical analysis revealed highly significant effects (*p* ≤ 0.01) of genotype, salinity level, and their interaction on the K^+^/Na^+^ ratio in soybean plants. The K^+^/Na^+^ ratio decreased dramatically with increasing salinity in both genotypes. The highest ratios were observed under control (154 in YAKARTA; 79.5 in POCA) and 25 mM NaCl conditions (151 in YAKARTA; 78.3 in POCA), with no significant differences between these two salt levels. In contrast, the lowest ratios occurred under severe salinity stress (100 mM NaCl), where values dropped to 0.27 in YAKARTA and 0.22 in POCA, indicating significant Na^+^ accumulation and disrupted ion balance. Across all salt levels, YAKARTA consistently maintained a significantly higher K^+^/Na^+^ ratio (61.8) than POCA (32.2) (Table 4), demonstrating better selective uptake and compartmentalization of potassium relative to sodium. This trait is commonly associated with salt tolerance, suggesting that YAKARTA possesses a more efficient ion regulation mechanism under salinity stress.

### 3.8. Ascorbic Acid Content (µg g^−1^)

**Figure 4:**
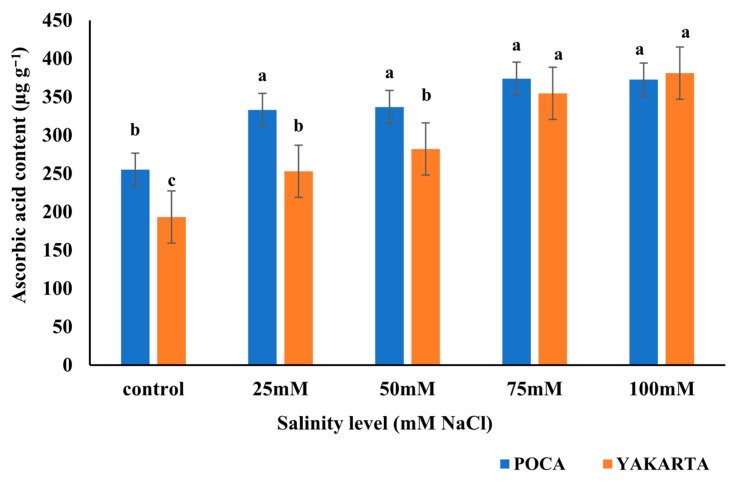
Effect of NaCl-induced salinity stress on ascorbic acid (µg g^−1^) content of two soybean genotypes (n = 3, ±s.e.). Different lowercase letters indicate significant differences (*p* ≤ 0.01) for the interactions between salinity levels and genotypes.

Statistical analysis revealed significant (*p* ≤ 0.01) effects of genotype, salinity level, and their interaction on ascorbic acid (AsA) content. A significant main effect of genotype was observed for AsA content (*p* < 0.01). Averaged across all salinity levels, POCA accumulated significantly more AsA (334 µg g^−1^) than YAKARTA (293 µg g^−1^), suggesting a constitutively stronger or more inducible antioxidant response. This overall trend is reflected in Figure 4, where POCA shows higher AsA values than YAKARTA at most individual salinity levels. Ascorbic acid levels increased significantly with salinity, reaching peak values at 100 mM (376.90 µg g^−1^) and 75 mM (364.30 µg g^−1^) NaCl, with no significant difference between the two. Notably, even under control (non-saline) conditions, POCA exhibited a significantly higher AsA content (255.10 µg g^−1^) than YAKARTA (193.30 µg g^−1^). In the interaction analysis, both genotypes showed significantly higher AsA levels at 75 and 100 mM NaCl. Additionally, POCA maintained significantly elevated AsA content at 25 and 50 mM NaCl (333.10 and 336.90 µg g^−1^, respectively), while YAKARTA showed its lowest value under control conditions (197.30 µg g^−1^) (Figure 4). These findings suggest that the greater accumulation of ascorbic acid (AsA) in POCA may reflect a stress-induced antioxidant response, likely triggered by its higher sensitivity to salinity. The elevated AsA levels may represent a compensatory mechanism aimed at mitigating the oxidative damage caused by salt-induced stress in this more susceptible genotype.

### 3.9. Hydrogen Peroxide Content (µmol g^−1^ Fresh Weight)

**Figure 5:**
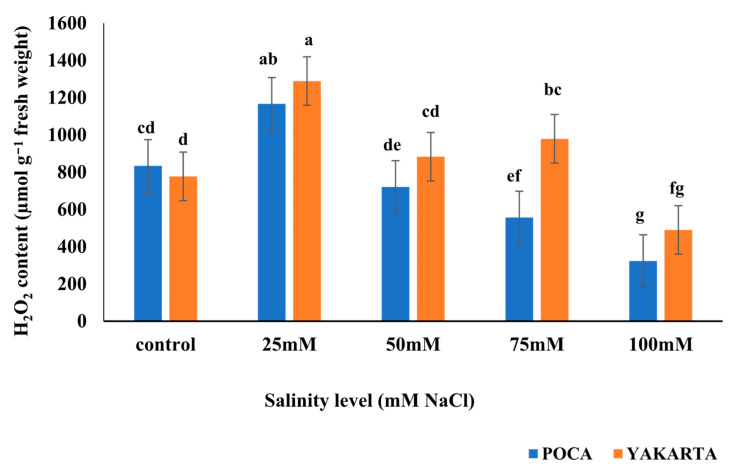
Effect of NaCl-induced salinity stress on hydrogen peroxide content (µmol g^−1^ fresh weight) of two soybean genotypes (n = 3, ±s.e.) under hydroponic conditions. Different lowercase letters indicate significant differences (*p* ≤ 0.01) for the interactions between salinity levels and genotypes.

Results revealed significant effects (*p* ≤ 0.01) of genotype, salinity level, and their interaction on hydrogen peroxide (H_2_O_2_) content in soybean leaves. YAKARTA showed significantly higher H_2_O_2_ accumulation (833.80 µmol g^−1^ fresh weight) than POCA (720.20 µmol g^−1^ fresh weight), suggesting a more intense oxidative response. Across treatments, H_2_O_2_ content peaked at 25 mM NaCl (1228.00 µmol g^−1^ fresh weight) and was lowest under 100 mM NaCl (406.50 µmol g^−1^ fresh weight). No significant differences were observed among control, 50, and 75 mM treatments, which recorded intermediate H_2_O_2_ levels (805.50, 801.90, and 767.90 µmol g^−1^ fresh weight, respectively). Interaction analysis showed that the highest H_2_O_2_ accumulation occurred in YAKARTA at 25 mM NaCl (1289.00 µmol g^−1^ fresh weight), followed by POCA at the same level (1167.00 µmol g^−1^ fresh weight)**.** In contrast, the lowest H_2_O_2_ levels were recorded in both genotypes at 100 mM NaCl, with POCA at 323.00 µmol g^−1^ fresh weight and YAKARTA at 490.00 µmol g^−1^ fresh weight, with no significant difference between them (Figure 5).

## 4. Correlation Analysis

**Figure 6:**
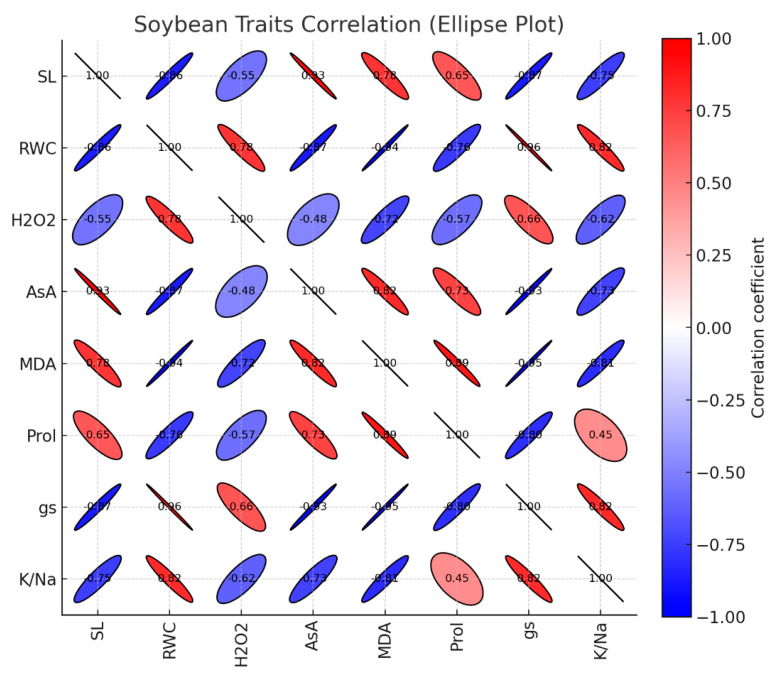
Ellipse-style correlation plot for the soybean traits, strong positive correlations form narrow red diagonals, strong negatives form narrow blue diagonals, and near-zero correlations appear as almost circular ellipses.

Correlation analysis of physiological and biochemical parameters in soybean under salinity stress revealed a strong interrelationship, underscoring the integrated nature of osmotic and oxidative stress adaptation. These associations are visualized in the ellipse correlation plot, where the shape and color intensity of each ellipse denote the strength and direction of the relationship between traits (Figure 6). A pronounced negative correlation was observed between solute leakage (SL) and relative water content (RWC) (r = −0.86**), indicating that genotypes capable of maintaining tissue hydration effectively minimize membrane damage. This aligns with the established interplay between cellular water status and membrane integrity, where dehydration promotes lipid peroxidation and subsequent ion leakage [45,46]. Furthermore, SL demonstrated significant negative correlations with both stomatal conductance (g_s_) (r = −0.87) and the K^+^/Na^+^ ratio (r = −0.75**). This pattern suggests that genotypes with superior stomatal regulation and ionic homeostasis are more proficient at preserving membrane structural and functional integrity under saline conditions [47,48].

Relative water content (RWC) demonstrated strong positive correlations with stomatal conductance (g_s_; r = 0.96**) and the K^+^/Na^+^ ratio (r = 0.82**) (Figure 6), underscoring that genotypes which maintain superior hydration are also more effective at sustaining photosynthetic gas exchange and ionic homeostasis [49]. Conversely, RWC was strongly and negatively correlated with key oxidative stress markers, including hydrogen peroxide (H_2_O_2_; r = −0.78**) and malondialdehyde (MDA; r = −0.94**) (Figure 6). This inverse relationship suggests that a preserved water status plays a critical role in attenuating oxidative damage, likely by preventing the conditions that lead to rampant ROS production and/or by supporting a robust antioxidant system [50]. A distinct pattern was observed for ascorbic acid (AsA), which correlated positively with MDA (r = 0.82**) and proline (r = 0.73**) but negatively with g_s_ (r = −0.93**) and the K^+^/Na^+^ ratio (r = −0.73**) (Figure 6). This profile implies that the accumulation of AsA is a responsive mechanism that is intensified under scenarios of significant oxidative stress and ionic disruption, conditions often precipitated by severe stomatal closure and compromised physiological function [51]. Proline accumulation exhibited a strong positive correlation with malondialdehyde (MDA) levels (r = 0.89) (Figure 6), indicating that its biosynthesis is co-induced with the progression of oxidative membrane damage. This coordinated response suggests that proline accumulation is a core component of the integrated defense against salinity-induced oxidative stress. These findings corroborate established models in which proline synthesis is upregulated in parallel with reactive oxygen species (ROS) generation, fulfilling multifaceted roles as a compatible solute for macromolecular stabilization, a direct scavenger of free radicals, and a key contributor to cellular redox homeostasis [52,53].

In summary, the correlation network reveals that salinity tolerance in soybean is defined by a synergistic suite of traits: the maintenance of high relative water content, stomatal conductance, and potassium-to-sodium ratio, coupled with low levels of solute leakage and oxidative stress markers (H_2_O_2_ and MDA). Genotypes coordinating this physiological profile, such as YAKARTA, demonstrate a superior capacity to preserve cellular integrity and mitigate oxidative damage. Consequently, the ellipse correlation plot serves as an integrative framework, visually synthesizing the core mechanisms, such as osmotic adjustment, ionic homeostasis, and oxidative defense, that underpin salinity resilience.

## 5. Principal Component Analysis (PCA)

**Figure 7:**
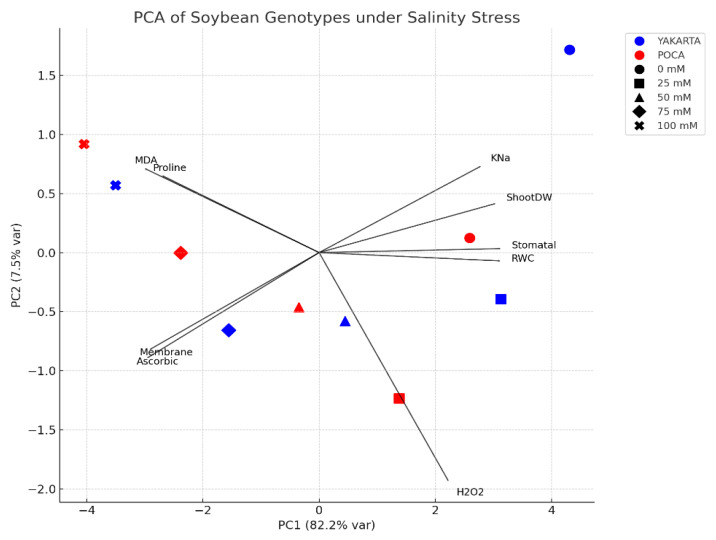
Principal component analysis of physiological and biochemical traits in soybean genotypes (YAKARTA and POCA) across salinity gradients.

Principal component analysis (PCA) revealed a clear physiological separation driven by salinity (Figure 7). PC1, which accounted for the majority of variance, was positively loaded with oxidative stress markers (malondialdehyde [MDA], hydrogen peroxide [H_2_O_2_]) and the osmoprotectant proline, indicating their coordinated increase under salt stress. Conversely, traits such as relative water content (RWC), stomatal conductance, K^+^/Na^+^ ratio, and shoot dry weight loaded negatively on PC1, confirming their suppression under high salinity. The PCA biplot effectively distinguished treatments: control and mild salinity (0–25 mM NaCl) clustered with traits for water and ionic homeostasis, while severe salinity (75–100 mM NaCl) associated with oxidative damage and antioxidant responses. Genotypic variation was evident, with YAKARTA positioned near traits for membrane stability and ionic homeostasis, indicative of a tolerant genotype. In contrast, POCA was associated with elevated proline, MDA, and H_2_O_2_, reflecting a more pronounced oxidative stress response and less efficient tolerance mechanism, consistent with known markers of salt sensitivity [54,55]. In summary, PCA effectively delineated the multivariate nature of salinity tolerance in soybean, revealing a genotype-dependent interplay between ionic regulation, osmotic adjustment, and oxidative defense. The superior performance of YAKARTA was associated with maintained ionic homeostasis (K^+^/Na^+^ ratio) and water status (RWC), indicative of a preventative tolerance mechanism. In contrast, POCA’s response is characterized by the strong induction of proline and antioxidants, which reflected a reactive defense against more severe cellular damage. These findings underscore membrane stability, ionic balance, and oxidative mitigation as cornerstone traits for salinity tolerance, providing a physiological framework for future breeding programs aimed at enhancing soybean resilience under salt stress.

## 6. Discussion

### 6.1. Morpho-Physiological and Biochemical Responses to Salinity Stress

This study provides novel insights into the early vegetative (V1–V4) responses of soybean genotypes to NaCl-induced salinity within a hydroponic system. Early-stage physiology during seedling establishment remains an underexplored yet critical window for determining later stress resilience. The controlled hydroponic setup allowed clear resolution of genotypic differences without soil-based confounding factors. Two contrasting patterns emerged: YAKARTA exhibited a coordinated suite of tolerance-related mechanisms, whereas POCA displayed physiological signatures characteristic of salt sensitivity.

#### 6.1.1. Shoot Growth, Biomass Decline, and Osmotic Constraints

The progressive reduction in shoot dry weight across salinity levels aligns with classical models of salt-induced osmotic stress, which reduce leaf turgor, inhibit stomatal conductance, suppress photosynthesis, and ultimately limit carbon assimilation. Similar reductions have been widely reported in soybean and other glycophytes, including in cultivars studied by Essa [22], Phang et al. [30], where salinity rapidly curtails shoot elongation and biomass accumulation during early growth. The consistently higher biomass of YAKARTA under moderate salinity (Figure 1) indicates greater maintenance of cell expansion and metabolic function, comparable to tolerant genotypes described by Phang et al. [30] and Amirjani [23].

#### 6.1.2. Osmotic Adjustment and Proline Accumulation

The ability of YAKARTA to maintain relative water content under increasing NaCl highlights efficient osmotic adjustment (Table 1). This was strongly supported by increased proline accumulation (Table 2), a feature commonly associated with stress tolerance in soybean. Multiple studies—including Hasanuzzaman et al. [56] and Sairam et al. [57]—identify proline as a multifunctional molecule contributing to osmoprotection, stabilization of proteins and membranes, and scavenging of reactive oxygen species (ROS). The pattern observed in this study mirrors findings by Rana et al. [58], where tolerant genotypes accumulated proline earlier and more strongly than sensitive ones. The lower proline accumulation in POCA (Table 2) is consistent with susceptibility, indicating insufficient osmotic buffering under salt stress.

#### 6.1.3. Membrane Integrity and Oxidative Stress: Contrasting Genotypic Behavior

Oxidative stress clearly differentiated the two genotypes. POCA accumulated significantly higher levels of MDA and solute leakage (Figure 3; Table 3), signaling advanced membrane lipid peroxidation. Similar patterns have been observed in sensitive soybean cultivars [59], where excess ROS overwhelms antioxidant defenses, leading to irreversible structural damage. In contrast, YAKARTA exhibited markedly lower MDA accumulation (Figure 3), indicating stronger antioxidant protection and membrane stability. This aligns with reports by Hasanuzzaman et al. [54], who found that tolerant soybean genotypes maintain membrane integrity through robust ROS-detoxifying systems. The elevated ascorbic acid (AsA) observed in YAKARTA (Figure 4), is consistent with enhanced activity of enzymatic and non-enzymatic antioxidants, similar to the responses described in tolerant soybean lines by Nounjan et al. [59]. This suggests that YAKARTA activates a more effective ROS-scavenging network compared to POCA.

#### 6.1.4. Hydrogen Peroxide Dynamics: A Biphasic Response Pattern

One of the most intriguing findings was the reduction in H_2_O_2_ concentration at the highest salinity level (100 mM) (Figure 5), despite the concurrent peak in MDA (Figure 3). This inverse pattern suggests a threshold effect in ROS metabolism, where extreme stress disrupts—not enhances—ROS production. As widely described in plant stress physiology, severe abiotic stress can cause a collapse of cellular metabolism, leading to impaired chloroplast and mitochondrial function, the primary sites of regulated H_2_O_2_ generation [60,61]. At lethal salinity, extensive membrane damage likely reduces compartmentalization and destabilizes electron transport processes, thereby limiting the production of H_2_O_2_ [62,63]. Simultaneously, the release of phenolic compounds and other antioxidants from damaged tissues may non-enzymatically quench residual H_2_O_2_ or interfere with its detection [51,64]. Accordingly, the low H_2_O_2_ levels observed at 100 mM NaCl should be interpreted as an indicator of functional failure of ROS-generating pathways rather than enhanced detoxification, consistent with the high MDA accumulation and extensive oxidative membrane damage. In contrast, the moderate H_2_O_2_ accumulation observed in YAKARTA under 25–50 mM NaCl (Figure 5), represents an adaptive oxidative burst, known to activate stress signaling and antioxidant defenses [65,66], which helps maintain membrane integrity and physiological performance. POCA’s higher H_2_O_2_ levels at moderate salinity suggest dysregulated ROS metabolism and insufficient detoxification, contributing to its greater oxidative damage.

Overall, these results underscore the context-dependent role of H_2_O_2_: in tolerant genotypes like YAKARTA, moderate ROS accumulation acts as a signaling cue to activate defense mechanisms, while in sensitive genotypes like POCA, excessive ROS indicates loss of regulatory control and contributes to oxidative damage. This mechanistic insight clarifies the apparently counterintuitive observation that YAKARTA can exhibit moderate H_2_O_2_ levels while maintaining superior physiological performance under salinity stress.

#### 6.1.5. Ion Homeostasis: Central Role of the K^+^/Na^+^ Ratio

Ion balance was a decisive physiological marker in this study. YAKARTA’s maintenance of a significantly higher K^+^/Na^+^ ratio (Table 4), is consistent with mechanisms of salt tolerance described in soybean, wheat, and rice. Salt-tolerant genotypes typically restrict Na^+^ loading into shoots, enhance selective K^+^ uptake, and maintain optimal cytosolic K^+^ levels to sustain enzyme activity. This pattern parallels findings by Phang et al. [30], Haque et al. [67], and Chen et al. [68], who identified K^+^/Na^+^ regulation as a key determinant of salinity resilience. POCA showed impaired K^+^ retention and sodium exclusion, reflecting compromised function of transporters such as HKT1, NHX1, or SOS1-type antiporters, which have been implicated in salt tolerance pathways in soybean [69,70]. The collapse of ionic balance contributed directly to POCA’s heightened oxidative damage and reduced biomass.

### 6.2. Integrated Interpretation into Salinity Tolerance Mechanisms

Our integrated interpretation demonstrates that salinity tolerance in soybean is a complex, multigenic trait involving coordinated morpho-physiological and biochemical processes. The tolerant genotype YAKARTA exhibited a preventative and well-regulated strategy, maintaining water status, preserving ionic balance, and minimizing oxidative injury. These attributes reflect a robust stress-avoidance mechanism that limits the progression of cellular damage. In contrast, the sensitive genotype POCA relied on a reactive and largely compensatory response, characterized by elevated oxidative stress markers, reduced K^+^/Na^+^ selectivity, and late proline accumulation, patterns typical of genotypes unable to maintain early homeostatic control under salinity.

Principal component analysis (PCA) further supported these physiological distinctions, revealing that the combination of osmotic regulation, ionic homeostasis, and antioxidant defense forms a unified tolerance network that explains most of the phenotypic variation between genotypes. This integrative pattern positions YAKARTA as a physiologically resilient genotype and provides a strong conceptual framework for breeding programs aiming to enhance salt tolerance in soybean.

Overall, YAKARTA’s tolerance can be interpreted as the convergence of three synergistic physiological strategies:

(1) Superior Osmotic Adjustment: YAKARTA effectively maintained relative water content (RWC) and accumulated higher proline concentrations, supporting osmotic balance, cellular hydration, and metabolic stability under increasing NaCl levels.

(2) Enhanced ROS Management and Membrane Protection: Lower levels of MDA, H_2_O_2_, and solute leakage indicate strong control of oxidative stress. The higher ascorbic acid (AsA) content in YAKARTA reflects a more efficient antioxidant defense, preventing membrane lipid peroxidation and preserving cellular integrity.

(3) Robust Ion Homeostasis: YAKARTA sustained a substantially higher K^+^/Na^+^ ratio than POCA, highlighting its capacity to restrict Na^+^ influx and maintain essential K^+^ functions, which are critical processes for enzyme activity, protein synthesis, and growth under salt stress.

These three strategies align with the conceptual model proposed by Munns and Tester [45], which identifies osmotic tolerance, ion exclusion, and tissue tolerance as the foundational pillars of plant salinity resilience. In contrast, POCA exhibited the hallmarks of a sensitive genotype, including rapid dehydration, membrane destabilization, compromised antioxidant capacity, and uncontrolled ionic imbalance.

### 6.3. Future Research Directions

Future research should focus on validating the key tolerance markers identified here, including RWC, K^+^/Na^+^ ratio, and oxidative stress indicators under field conditions across diverse environments. A multi-omics approach, integrating transcriptomic, proteomic, and metabolomic profiles, is essential to delineate the genetic architecture and regulatory networks governing these physiological responses. Furthermore, studies must quantify the contribution of early-stage tolerance traits to final agronomic outcomes, such as biomass and seed yield. Concurrently, exploring agronomic strategies like nutrient management, biochar amendment, or exogenous osmoprotectant application could offer immediate pathways to mitigate yield loss in saline soils. Bridging this mechanistic understanding with practical interventions will accelerate the development of resilient soybean cultivars for salt-affected regions.

## 7. Conclusions

This study elucidates key physiological and biochemical determinants of early-stage salinity tolerance in soybean. The tolerant genotype YAKARTA maintained homeostasis through coordinated osmotic adjustment, antioxidant activity, and ion regulation, whereas the sensitive genotype POCA suffered oxidative damage and ionic disruption. By comparing these two contrasting genotypes, we identified several trait responses—including maintenance of relative water content and K^+^/Na^+^ ratio, proline accumulation, and oxidative damage (MDA)—that serve as promising candidates for further investigation as screening biomarkers. However, given that this study involved only two genotypes under controlled conditions, the applicability of these traits as robust, generalizable markers for breeding require validation across diverse genetic backgrounds and field environments. Nonetheless, these findings provide a mechanistic framework that highlights specific physiological and biochemical traits as high-priority targets for future efforts aimed at improving soybean salinity tolerance.

## Figures and Tables

**Table 1:** Effect of NaCl-induced salinity stress on relative water content (%) of two soybean genotypes under hydroponic conditions.

Salinity Level (mM NaCl)	YAKARTA	POCA	Mean
Control	94.25 ^aA^	89.83 ^aA^	92.04 ^A^
25	87.90 ^bA^	80.62 ^bB^	84.26 ^B^
50	75.18 ^cA^	66.43 ^cB^	70.80 ^C^
75	62.39 ^dA^	53.25 ^dB^	57.82 ^D^
100	57.83 ^eA^	44.35 ^eB^	51.09 ^E^
Mean	75.51 ^A^	66.97 ^B^	-
Variable	Genotypes	Salinity levels	Interaction
LSD (0.01)	9.75	4.78	6.77
CV (%)	3.85		

Different lowercase letters (a–e) within a column indicate significant differences (*p* ≤ 0.01) among NaCl-induced salinity stress treatments within and between genotypes (based on the genotype × NaCl interaction). Capital letters (A–E) indicate significant differences (*p* ≤ 0.01) among mean values for genotypes or NaCl levels, averaged across the other factor.

**Table 2:** Effect of NaCl-induced salinity stress on proline content (µmol g^−1^ fresh weight) of two soybean genotypes under hydroponic conditions.

Salinity Level (mM NaCl)	YAKARTA	POCA	Mean
Control	0.25 ^dA^	0.24 ^dA^	0.25 ^C^
25	0.45 ^dA^	0.35 ^dA^	0.40 ^C^
50	0.77 ^dA^	0.67 ^dA^	0.72 ^C^
75	14.0 ^bA^	7.76 ^cB^	10.9 ^B^
100	15.3 ^aA^	12.8 ^bB^	14.0 ^A^
Mean	6.15 ^A^	4.36 ^B^	-
Variable	Genotypes	Salinity levels	Interaction
LSD (0.01)	1.56	1.43	2.02
CV (%)	16.1		

Different lowercase letters within a column indicate significant differences (*p* ≤ 0.01) among NaCl-induced salinity stress treatments within and between genotypes (based on the genotype × NaCl interaction). Capital letters indicate significant differences (*p* ≤ 0.01) among mean values for genotypes or NaCl levels, averaged across the other factor.

**Table 3:** Effect of NaCl-induced salinity stress on membrane integrity (solute leakage) (%) of two soybean genotypes under hydroponic conditions.

Salinity Level (mM NaCl)	YAKARTA	POCA	Mean
Control	63.4 ^dA^	81.6 ^bcB^	72.5 ^D^
25	73.4 ^cA^	83.8 ^bB^	78.6 ^CD^
50	82.0 ^bcA^	85.2 ^abA^	83.6 ^BC^
75	85.9 ^abA^	90.9 ^abA^	88.4 ^AB^
100	91.2 ^abA^	94.2 ^aA^	92.7 ^A^
Mean	79.2 ^B^	87.1 ^A^	-
Variable	Genotypes	Salinity levels	Interaction
LSD (0.01)	5.84	6.80	3.52
CV (%)	4.85		

Different lowercase letters within a column indicate significant differences (*p* ≤ 0.01) among NaCl-induced salinity stress treatments within and between genotypes (based on the genotype × NaCl interaction). Capital letters indicate significant differences (*p* ≤ 0.01) among mean values for genotypes or NaCl levels, averaged across the other factor.

**Table 4:** Effect of NaCl-induced salinity stress on K^+^/Na^+^ ratio of two soybean genotypes under hydroponic conditions.

Salinity Level (mM NaCl)	YAKARTA	POCA	Mean
Control	154.00 ^aA^	79.50 ^bB^	117 ^A^
25	151.00 ^aA^	78.30 ^bB^	115 ^A^
50	2.41 ^cA^	2.12 ^cA^	2.27 ^B^
75	1.32 ^cA^	0.67 ^cB^	1.00 ^B^
100	0.27 ^cA^	0.22 ^cA^	0.24 ^B^
Mean	61.8 ^A^	32.20 ^B^	-
Variable	Genotypes	Salinity levels	Interaction
LSD (0.01)	3.52	3.54	7.03
CV (%)	8.53		

Different lowercase letters within a column indicate significant differences (*p* ≤ 0.01) among NaCl-induced salinity stress treatments within and between genotypes (based on the genotype × NaCl interaction). Capital letters indicate significant differences (*p* ≤ 0.01) among mean values for genotypes or NaCl levels.

## Data Availability

The datasets generated and analyzed during the current study are available from the corresponding author on reasonable request.

## Supplementary Materials

The following supporting information can be downloaded at: https://www.mdpi.com/article/10.3390/plants15010010/s1, Figure S1: Comparative shoot and root morphology of the sensitive genotype POCA (left) and the tolerant genotype YAKARTA (right) grown under increasing NaCl treatments (Control, 25, 50, 75, and 100 mM NaCl) in a hydroponic system. The images illustrate genotypic differences in whole-plant vigor, root system architecture, and visible symptoms of salt-induced damage across the salinity gradient.
