# Supplementary material for: Evaluation of Salinity Tolerance Potentials of Two Contrasting Soybean Genotypes Based on Physiological and Biochemical Responses
Source: Plants (Basel). 2025 Dec 19;15(1):10. doi: 10.3390/plants15010010 (PMC12787487; doi:10.3390/plants15010010)
Supplement: Supplementary file 1 [file plants-15-00010-s001.zip › plants-4022901-supplementary.pdf]

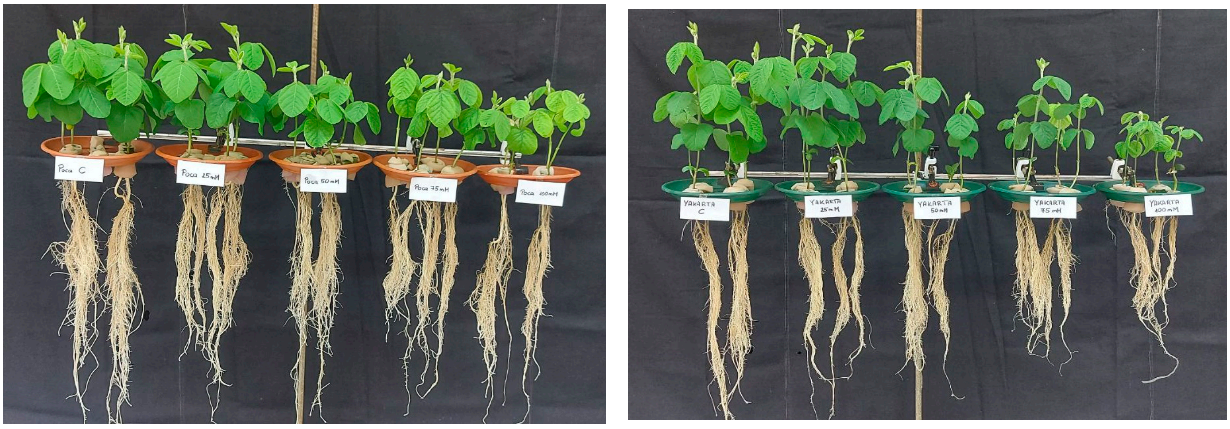

Figure S1. Comparative shoot and root morphology of the sensitive genotype POCA (**left**) and the tolerant genotype YAKARTA (**right**) grown under increasing NaCl treatments (Control, 25, 50, 75, and 100 mM NaCl) in a hydroponic system. The images illustrate genotypic differences in whole-plant vigor, root system architecture, and visible symptoms of salt-induced damage across the salinity gradient.
